# Intra-uterine Medication

**Published:** 1880-04-20

**Authors:** C. D. Palmer

**Affiliations:** Cincinnati, Ohio


### *INTRA-UTERINE MEDICATION.*

A Paper read before the Cincinnati Obstetrical Society.

BY C. D. PALMER, M.D., CINCINNATI, O.

There are many points connected with intra-uterine medication still *sub-judice*. Recently the subject was up for discussion before the British Medical Association, and also at the last meeting of the American Gynecological Society. This Society should compare its experiences.

It is chronic endo-metritis, some of its conditions or complications which, for the most part, call for intra-uterine medication. Reference is had here, not to medication exterior to the cervical canal, but to the corporeal cavity of the uterus.

Three varieties of chronic endo-metritis must be recognized :

1st. Cervical; 2d, general; 3d, corporeal.

This, undoubtedly, is also the order of frequency.

The propriety of medication of the cervical canal is, I believe, questioned by no one, but there are those who do object to, and never medicate the corporeal cavity of the uterus. By most gynecologists of to-day, intra-uterine medication (the term is used in its general sense, meaning the use of tents, curette, etc., as well as medicines), is practiced with varying frequency, some extending applications almost as often to a point above the os internum as below.

When this subject was being freely discussed in New York in 1870, and when some most important problems connected therewith were solved, a most decided impetus was given to the employment of this method of uterine treatment all over the country. Whether or not intra-uterine medication has met with its highest expectations, and with what dangers, with what results, can be obtained best by a free discussion, which, I trust, will be elicited here.

Are we warranted in medicating the corporeal cavity of the uterus, *when* and *under what circumstances*, *how*, and with *what agents ?*

It is almost unnecessary to remark, that where endo-metritis is purely cervical, the endometrium above the internal os should not be interfered with. Now, in general endometritis, the intensity of the disease may be at a point above or below the boundary line of the internal os. This will be determined by the degree of the presence of certain symptoms; menstrual aberration, as menorrhagia as to time, quantity and duration; certain forms of dysmenorrhœa; the character of the leucorrheal discharge; as well as certain signs: the patulous internal os, tender and dilated corporeal cavity, and the enlarged corpus uteri. This class of cases is largely confined to multiparæ, and follows a bad getting up after an abortion or parturition at term. Can such cases be treated by cervical medication, or is it necessary to extend the topical treatment to the fundus of the uterus?

My rule in practice has been as follows :

Where the disease is, seemingly, for the most part cervical, the menstrual function not seriously deranged, to trust to cervical treatment. In a majority of cases, it suffices, without intra-corporeal medication, the latter being adopted only in cases of failure of the former, and in the more severe types of the disease. My experience has been clear that cervical treatment in the way of depletion and medication, does, in many instances, exercise a most decided influence over the body of the uterus, both in its endometrium and parenchyma. Besides the relief of the cervical disease, and its reactive influence on the general health, cervical medication has a derivative effect on the disease beyond.

Simply then because the internal os has been sufficiently dilated to permit the passage of the sound or the cotton-wrap applicator, has not been the guide for the extension of the topical treatment to the fundus. If cervical treatment will answer equally well for many and the milder forms of chronic endometritis of a general character, why adopt the other with its increased dangers?

But there are rarer forms of general endometritis, the chief seat of which is the corporeal cavity. This is considerably dilated; the body of the organ may be enlarged; muco-purulent leucorrhœa and menorrhagia are present. A fungoid or cystic degeneration of the endometrium is usually the underlying condition. This kind of a leucorrhœa and menorrhagia will resist constitutional, as well as local cervical treatment, including dilatation. Intra-corporeal treatment is necessary, and even this, if confined to medication, frequently fails. The curette, moderately sharp, the end of which is tempered, the shank flexible, applied at first gently, afterwards, if well borne and necessary, more thooughly, has served me so many useful turns that its use seems indispensable in this class of cases. This hyperplastic condition of the endometrium with granulations is most common in the region of the cornua of the uterus. In many instances a strong solution of iodine is then applied. One curetting sometimes suffices; more often its repetition, from once per week to once per month, with the iodine applications, is required before a cure is effected.

As to the intra-uterine injections, these are sometimes employed to wash out the uterus, on account of septic discharges from a disintegrating fibroid, cancer, etc.; also in stubborn menorrhagia, to arrest the flow. Never have I encountered any serious symptoms even of the shortest duration, with my double current catheter, with which retention of the injected fluid and consequent pain or shock cannot occur.

I like the action of Lente's cotton-wrapped syringe, which secures thoroughness of application without a canula.

Tents usually are not required; if any, preference is given to the laminaria; the metallic dilator, for ordinary dilatation, has been found safe and sufficient.

A short cervical canula, fashioned much after the shape of Atthill's, sometimes is used when a more active agent, such as nitric acid, is applied to the fundus.

As to the selection of medical agents for application to the corporeal cavity, generally speaking, I have found nothing so satisfactory as iodine in strong solution, used with a flexible probe of silver, or with Lente's syringe. Iodine has proven to be a general alterative, a local stimulant to uterine contraction and to the blood vessels, thereby diminishing congestion. This, with a solution of chromic acid (water and acid equal parts), and pure nitric acid, are most of the agents used to this region of the uterus. Carbolic acid is largely reserved for disinfecting purposes. Medicated tents, as well as crayons and ointments containing medicines, have not proven satisfactory.

Nitrate of silver, either in solution or in crayon, I now never employ to the upper uterine cavity, because of the amount of pain and hemorrhage at times produced, and the tendency to contractions of the os. Nitric acid accomplishes all that the nitrate of silver possibly can do, and strange as it may appear, is infinitely less painful, creates little or no hemorrhage—in fine, is better and safer. Its use should be reserved to patulous conditions of the canal, and its contracting effects carefully watched.

I do not share the opinion of those who believe that the upper cavity of the uterus can be disturbed by instruments and medicines with as much safety and impunity as the cervical canal. The late distinguished Prof. Miller, who was probably the first in this country to institute the practice of intra-uterine medication, maintained that he could treat the upper cavity with as much familiarity and as little apprehension as the cervix. Again, one of our first gynecologists, speaking on the subject, says:

"I have come to the conclusion that he is the most successful gynecologist who is the most plucky, and that, no matter how severe or mild the treatment of uterine disorders, the percentage of accidents will be about the same"; a statement of doubtful propriety and of doubtful truth, if we are to accept the results of the treatment of all who practice in this field.

The upper uterine cavity cannot be interfered with at all times with impunity. All uterine treatment is attended with a certain amount of risk. Other things being equal, the further within the canal that treatment, the greater the risk. Thus, in point of safety, it makes a very considerable difference as to whether any interference is above or below the line of the internal os. Fortunately the greater the disease and longer its duration, the more profuse the secretions, conditions calling most for local treatment, the greater the degree of tolerance.

While then I favor topical medication to the corporeal cavity in certain instances, nay, even regard it as essential at times to success, nevertheless, the conviction has grown from increasing experiences that many cases of endo-metritis which heretofore have been treated as corporeal, at which point doubtless more or less of a congested state of that mucous membrane has existed, *might be*, *ought to be* treated purely as cervical, and this with less pain, greater safety, and an equal certainty of good results.

Bennett, to whom we owe a world of gratitude for his pioneer contributions in this department, was a firm believer in the cervical localization to a large degree of chronic uterine inflammations. To this end his treatment was directed. While, doubtless, he was in error in excluding the corpus uteri to the extent he did from complicity in the diseases of the cervix, the success which attended his efforts confirms my position.

Prof. Thomas, speaking on this subject at the last meeting of the American Gynecological Society, remarked that "intra-uterine medication carried above the os-internum should be given up as very hazardous, in many cases as very useless, and yielding disappointing results." This is certainly a very striking position for one who, doubtless, has resorted to this method very frequently, and has had every opportunity to test this question to its fullest extent.

Much of the ill-success attending intra-uterine medication is, aside from the neglect of securing proper cleanliness of the endo-metrium prior to the introduction of the medicament, or some other imperfect manner of application, due to the fact that the treatment has been too severe and too frequently repeated. If experience has taught us anything in this special field of gynecological practice in the past few years, it is to make intra-uterine medication less painful, safer, and limit its field of utility.

Endo-metritis complicated by versions and flexions as an antecedent or consequent condition, will, of course, be managed largely with reference to the causative relationship. Unfortunately, this can by no means be always satisfactorily determined.

Shall we treat the diseased endo-metrium with the uterus still in its faulty position, or, first correct the mal-position? Will the displacement be relieved after the circulation has been improved by topical treatment to the endo-metrium, or will the endometrical affection subside after the rectification of the displacement?

It is extremely difficult in our experience to establish a uniform rule of order of procedure. Retroversion and flexion usually follow the congested, enlarged, and relaxed conditions of the uterus after parturition at term, or an abortion. Here the displacement is secondary; but the supervention of this displacement not only aggravates, but perpetuates, and may otherwise render the former state incurable without a notification of this displacement.

Many cases of chronic catarrh of the inner uterus, with dysmenorrhœa or menorrhagia, are resultant on version, especially flexion. The same becomes speedily relieved by a successful attention alone to the displacement. Under such circumstances intra-uterine medication is not only unnecessary but harmful. But on the other hand, how often has it happened in practice that a prolapsed or retroverted uterus gradually returns to its original position by attention to the co-existent but antecedent endo-metritis and sub-involution? It is almost unnecessary to remind the members of the dangers connected with intra-uterine medication when there is co-existent, chronic, cellular, or peritoneal inflammations. He becomes most prudent who witholds interference with the uterine cavity in the not unfrequent class of cases complicated with obscure or latent cellulitis.

Unquestionably the most stubborn of all forms of intra-corporeal inflammation exists in the nulliparous uterus. The severity and duration of the local symptoms, seemingly, call for local treatment in unmistakable terms. But local treatment here, usually, utterly fails. At the bottom and within the backgrounds of these cases, there is a serious constitutional dyscrasia, the correction of which is essential to the permanency of relief. At best there can be a local improvement only commensurate with the general.—*Ob. Gazette.*

